# Community pharmacists’ practices and clinical reasoning towards hospital discharge prescription: a study using simulations and retrospective think-aloud methodology

**DOI:** 10.1007/s11096-025-01978-0

**Published:** 2025-08-26

**Authors:** Léa Solh Dost, Bertrand Guignard, Giacomo Gastaldi, Aveen Hasan Hamzo, Mathieu Nendaz, Marie-Claude Audétat, Marie P. Schneider

**Affiliations:** 1 School of Pharmaceutical Sciences, University of Geneva, Geneva, Switzerland; 2 Institute of Pharmaceutical Sciences of Western Switzerland, University of Geneva, Geneva, Switzerland; 3 Pharma24, Academic Community Pharmacy, Geneva, Switzerland; 4 Pharmacy, Geneva University Hospitals, Geneva, Switzerland; 5 Division of Endocrinology, Diabetes, Hypertension and Nutrition, Department of Medicine, Geneva University Hospitals, Geneva, Switzerland; 6 Unit of Development and Research in Medical Education (UDREM), Faculty of Medicine, University of Geneva, Geneva, Switzerland; 7 Department of Medicine, Geneva University Hospitals, Geneva, Switzerland; 8 University Institute for Primary Care, Faculty of Medicine, University of Geneva, Geneva, Switzerland

**Keywords:** Clinical reasoning, Community pharmacist, Continuity of patient care, Decision making, Medication Reconciliation, Patient simulation

## Abstract

**Background:**

The roles of community pharmacists have evolved from dispensing medications to clinical decision makers. This shift requires a clearer understanding of pharmacists’ clinical reasoning. Managing hospital discharge prescriptions requires analytical reasoning to ensure patient safety through medication reconciliation and patient education.

**Aim:**

This study assessed community pharmacists’ practices and their clinical reasoning towards hospital discharge prescriptions.

**Method:**

This mixed-method study consisted of two phases. First, community pharmacists participated in a simulated encounter in their pharmacy, where a patient presented a discharge prescription. Their practices and the structure of the encounter were assessed using a structured checklist of practices adapted from the MEDICODE checklist. Following the simulation, participants verbalised their thought processes in a retrospective think-aloud session. These semi-structured interviews were transcribed and analysed using both inductive and deductive qualitative methods. Charlin et al.’s model was used to assess clinical reasoning, while the Calgary–Cambridge model evaluated communication structure.

**Results:**

Among 14 participating pharmacists, 13 performed medication reconciliation, and 10 contacted the simulated prescriber to address discrepancies. While most provided adherence aids, only seven assessed non-adherence, and five actively collaborated with the patient. Pharmacists exhibited diverse interview structures, often revisiting previous discussion points. Clinical reasoning misconceptions, such as assumptions or premature closure, were observed at multiple stages of the clinical reasoning process.

**Conclusion:**

Community pharmacists demonstrate strong medication-related skills but face challenges in clinical reasoning for discharge prescriptions. Clinical reasoning training, semi-structured consultations, and greater patient engagement would help tailor and improve post-discharge care.

**Supplementary Information:**

The online version contains supplementary material available at 10.1007/s11096-025-01978-0.

## Impact statements

- Community pharmacists show strong medication-related skills in managing hospital discharge prescriptions when participating in an on-site simulation, but face challenges in patient-related tasks.
- Adopting consistent, evidence-based frameworks that maintain flexibility for individual patient needs and preferences would strengthen the currently unstandardised practices of community pharmacists.
- Community pharmacists demonstrate analytical clinical reasoning when managing medication-related issues.
- Their metacognitive awareness demonstrates an ability to reflect on practice and recognise potential biases; however, targeted training in clinical reasoning is needed to optimise decision-making further.

## Introduction

Community pharmacists and their teams are among the most accessible healthcare professionals [1]. These frequent contacts provide opportunities to educate and support patients with their medications [2]. As pharmacotherapy and patient health issues become more complex, community pharmacists are shifting from traditional dispensing roles to becoming medication experts and key decision-makers in comprehensive care [2, 3]. Such practices demand clinical reasoning skills, enabling them to make informed and evidence-based decisions [3, 4]. However, this shift also requires a better characterisation of community pharmacists’ role and responsibilities within the healthcare team [4, 5].

Clinical reasoning is increasingly recognised in pharmacy practice as a core cognitive process for assessing and managing patient care [3–6]. It involves gathering and interpreting patient information, weighing benefits and risks, and considering patient preferences to develop a shared management plan. It is a dual process, during with practitioners shift between intuitive (rapid, pattern-based) and analytical (deliberate, systematic) reasoning depending on case complexity and experience [7, 8]. Both processes are prone to error: intuitive reasoning can be affected by cognitive biases or clinical reasoning misconceptions, while analytical reasoning may be impacted from faulty logic or mental overload. In practice, clinicians combine both approaches to optimise decisions [9, 10].

Various clinical reasoning models exist to describe clinical reasoning steps, such as the Pharmacists’ Patient Care Process in pharmacy or Wright’s clinical decision model in pharmacy practices [11, 12]. Charlin et al.’s clinical reasoning process is a comprehensive and generic model that captures the clinical reasoning process of all healthcare professionals [13, 14]. A relatively small number of qualitative studies have captured community pharmacists’ clinical reasoning by looking at over-the-counter and prescription dispensing [15, 16]. A scoping review showed analytical and intuitive approaches to pharmacists’ clinical reasoning, with a predominantly analytical approach when determining the medication appropriateness of prescribed medications [5].

The transition from inpatient to outpatient care presents significant risks to patient safety due to challenges in the continuity of care and medication management [17–20]. In the context of hospital discharge, community pharmacists’ responsibilities extend beyond dispensing medications and include ensuring continuity of care, optimising therapeutic outcomes, and preventing drug-related problems [21, 22]. Community pharmacists are positioned to bridge the therapeutic gap between hospital and outpatient care [21, 23]. However, their roles and responsibilities at transition are not well-defined [24, 25]. Managing and dispensing hospital discharge prescriptions is a multifaceted process that requires community pharmacists to manage complex prescriptions [26, 27]. Due to the frequent changes in medication regimens and patients’ limited understanding of their treatments, community pharmacists must apply strong analytical reasoning to ensure patient safety during medication reconciliation. To our knowledge, no research has examined how community pharmacists apply clinical reasoning in the management and dispensing of hospital discharge prescriptions.

### Aim

The aim of this study was to assess community pharmacists’ practices and their clinical reasoning towards hospital discharge prescriptions.

### Ethics approval

The University Commission for Ethical Research in Geneva (CUREG-MM-2023–05-67) sought and granted ethics committee approval. This qualitative study followed the Consolidated Criteria for Reporting Qualitative Research (COREQ).

## Method

### Study design

This mixed study consisted of two parts: a simulated patient‒pharmacist encounter based on a case scenario (quantitative), followed by a retrospective think-aloud interview (qualitative). This combined approach provides both direct observation of practice, as the in-situ simulation ensures a realistic assessment of practices as well as a deeper understanding of the cognitive processes underlying their actions, as participants verbalise their reasoning and thought processes during the interview [28].

### Study location and recruitment

The participants were community pharmacists working in the canton of Geneva, Switzerland. This study was conducted from August to October 2023. Participants were recruited through the Geneva local professional association via email and during a professional meeting and selected through purposive sampling based on experience (years of practice), location (rural or metropolitan; independent or chain pharmacies), and position (staff pharmacist, pharmacy manager, or holding an additional degree). LS, a PhD Student and community pharmacist, knew 6 out of 14 participants through professional interactions. Interested participants provided their informed written consent before participation and could revoke it at any time. The participants were informed of the methodology and timing of the encounter but not about the case scenario.

Two patient partners (PP) with type 2 diabetes and at least two comorbidities, who had experienced hospital discharge, were recruited through the Hospital Patient Partner Platform to realistically simulate the patient scenario.

### Procedures

#### Simulated encounter

The PP entered the pharmacy and presented the discharge prescription to the pharmacist, who was instructed to process it per routine practice. A trained simulated prescriber was available by phone to respond if the pharmacist chose to contact them.

The case scenario (Table 1) involved a polypharmacy patient with type 2 diabetes recently discharged from the hospital. Designed to reflect common post-discharge challenges [24, 25, 29–34], the scenario assessed how pharmacists managed medication reconciliation and review as well as their approach to medication nonadherence, self-management difficulties, and knowledge gaps at treatment initiation.

**Table 1:** Case description

Case description The patient was hospitalised for uncomplicated hyperglycaemic decompensation for four days. He/she has a history of transient ischaemic attack, type 2 diabetes, and depression. He/she usually has difficulties taking medication and has a lack of knowledge about empagliflozin. He/she is interested in having information on side effects	
Prescription *(DCI, dosage, instructions)* Metformin, 1000 mg, morning and evening Empagliflozin, 10 mg, once a day Escitalopram (original), 20 mg, morning Valsartan, 160 mg, morning Pravastatin, 40 mg, evening	
Medication history *(given only if requested by the pharmacist, as a treatment card)* Metformin, 500 mg, morning and evening Escitalopram (generic) 20 mg, morning Valsartan, 80 mg, evening Pravastatin, 40 mg, evening Acetylsalicylic acid, 100 mg, morning	

#### Training of simulated patients

The patient partners received three hours of training on study objectives, methods, and the scenario, followed by mock simulations. Their feedback refined the scenario. They were instructed to respond only when prompted. A pilot with a nonparticipating pharmacist ensured standardised behaviour and language.

#### Retrospective think-aloud

Immediately following the simulation, pharmacists’ clinical reasoning was assessed using a retrospective think-aloud interview. Each participant was asked to verbalise their thoughts, decision-making processes, and reasoning at each key step. A semi-structured interview guide (see Supplementary Material 1) was used to ensure consistency across interviews and to prompt discussion on specific decision points and encountered challenges.

### Model-based approach to pharmacist-patient interaction

Pharmacists’ practices were analysed based on the MEDICODE classifications [35], a validated coding tool for medication-related communication. The checklist was adapted to Swiss pharmacy services and prescription validation criteria from the Swiss pharmacy state diploma OSCE [36] (see Supplementary Material 2).

The Calgary-Cambridge framework, adapted for pharmacist-patient encounters, analysed the structured approach in four steps (initiating, gathering information, explanation/planning, closing) and two transversal principles (structure, relationship) [37].

The Charlin et al. clinical reasoning model described pharmacists’ reasoning through six dynamic, nonlinear subprocesses with feedback loops [13]: “identify early cues,” “determine the objectives of the encounter,” “categorise for the purpose of action,” “implement alternative strategies,” “implement purposeful action,” and “evaluate the results.” These steps are further supported by two overarching processes: “organising knowledge for clinical action” and “regulating one’s cognitive processes.”

### Data collection and analysis

#### Simulated encounters

The simulated encounter was video- and audio-recorded. The practices checklist (Supplementary Material 2) was completed by AH, a master’s student, and independently verified by LS through review of the video recording. Inter-rater reliability was ensured by double-scoring all checklists and resolving any discrepancies through discussion and consensus.

The simulated encounters were quantitatively analysed for the presence or absence of items on the checklist, and the structure of the encounters was mapped and compared to the Calgary–Cambridge model [37].

#### Retrospective think aloud

Audio recordings were transcribed AH and checked for accuracy by LS. The retrospective think-aloud transcripts were analysed by LS, MS, a community pharmacist specialised in research in medication adherence, a psychologist specialised in research in medical education (MCA), and a hospital clinical pharmacist (BG). LS, MS, and MCA were trained in qualitative analysis. MCA and BG had expertise in the field of clinical reasoning. After analysing 11 interviews, three additional interviews were conducted to ensure data saturation and capture diverse professional experiences. Thematic saturation was reached when no new codes or themes emerged [38]. To enhance rigor and consistency, the research team conducted double coding until consensus, met regularly to validate a coding framework and systematically discussed the analysis to achieve consensus.

The thematic analysis was deductive (theory-driven) and inductive (data-driven) using MAXQDA® (2018.2). Deductive analyses from the clinical reasoning model outlined by Charlin et al. and the medical encounter Calgary–Cambridge model were subsequently performed [13, 37]. Data that did not fit within the predefined frameworks were coded using an inductive approach, allowing new themes and categories to emerge directly from the data. In a subsequent step, clinical reasoning misconceptions and biases identified during the thematic analysis were categorised using the terminology and classification of a published framework [39, 40].

### Data management

Recordings were securely stored on the University of Geneva server, with pseudonymizing of the transcripts to ensure confidentiality. Video and audio recordings were destroyed after analysis and publication. Video and audio files were destroyed after analysis. Only pharmacists and patient partners were videotaped to protect patient confidentiality.

## Results

### Pharmacists’ characteristics

Fourteen pharmacists from 11 pharmacies participated in the study from August to October 2023. The patients’ characteristics are presented in Table 2. The median duration of the simulated encounters was 20 min (IQR: 18–21; min–max: 9–38) and 17 min (IQR: 16–20) for the retrospective think-aloud interviews.

**Table 2:** Pharmacists characteristics

Pharmacists’ characteristics	N
Pharmacists	14
Gender	
Male	5
Female	9
Professional experience (years)	
1–10	7
11–20	3
21–30	3
> 30	1
Pharmacy manager	6
Additional degree	
Postgraduate degree*	2
PhD	1
Participating pharmacies	11
Type of pharmacies	
Chain pharmacy	5
Independent pharmacy	6

*Continuing education leading to the Swiss Federation (FPH) certificate required since 2019 to become a pharmacy manager

### Pharmaceutical practices

Twelve encounters were held at the counter, and two pharmacists offered the patient to move to a consultation room (a separate room to provide pharmaceutical care). Pharmacists raised and discussed a median of 31 topics (IQR: 27;35; min–max: 15–60) during the simulated encounter. Table 3 summarises the topics that the pharmacists discussed at least once during the encounter (full results in Supplementary Material 3).

**Table 3:** Summary of topics discussed at least once by pharmacists during the encounter

Decisive topics discussed at least once	N pharmacists
Creating a patient file	14
Checking and completing medication history	13
Identifying medication discrepancies	
Introduction of a new medication	12
Omission of a medication	12
Identification of a generic switch	7
Medication reconciliation intervention resulted in	
Contacting the prescriber	10
Dispensing without medical approval	2
No dispensing of the medication	2
Dispensing medications	
Labelling medication boxes with intake instructions	14
Explanation of the instructions for use	14
Having to order a medication	8
Patient medication knowledge	
Assessment of the patient’s understanding	2
Identification of the patient’s need for information	3
Provision of information on medication indication	12
Provision of information on side effects	8
Patient medication management and adherence	
Assessment of the degree of (non)adherence	7
Consideration of patients’ opinions and resources	5
Discussion of adherence aids	11
Monitoring and closing	
Checking the appropriate self-management/understanding of additional monitoring measures (e.g. blood glucose)	9
Checking understanding by asking patients to state in their own words what they understood (teach-back method)	2

#### The structure of the encounter

**Fig. 1:**
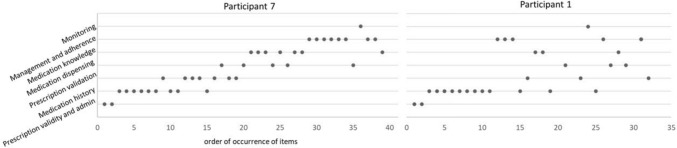
Visual representation of the structure of the encounters (Participant 7 and Participant 1)

Supplementary Material 4 provides a visual representation of the structure of each encounter. Three distinct patient-pharmacist interview structures emerged. Four followed a thematic approach (P4, 7, 9, 10), aligning with key pharmacy practice areas, as illustrated by Participant 7 in Fig. 1. Six pharmacists (P1, 3, 6, 10, 11, and 13) initiated the encounter by opening and reviewing the patient pharmacy file and medication reconciliation, then proceeded without a clear, structured flow, often revisiting items based on patient input. A third group (P2, 5, 8, 12) showed no discernible structure, as shown by Participant 1 in Fig. 1. None of the pharmacists fully aligned with the Calgary–Cambridge Medical Encounter Model.

### A model combining community pharmacists’ clinical reasoning and the Calgary–Cambridge model

**Fig. 2:**
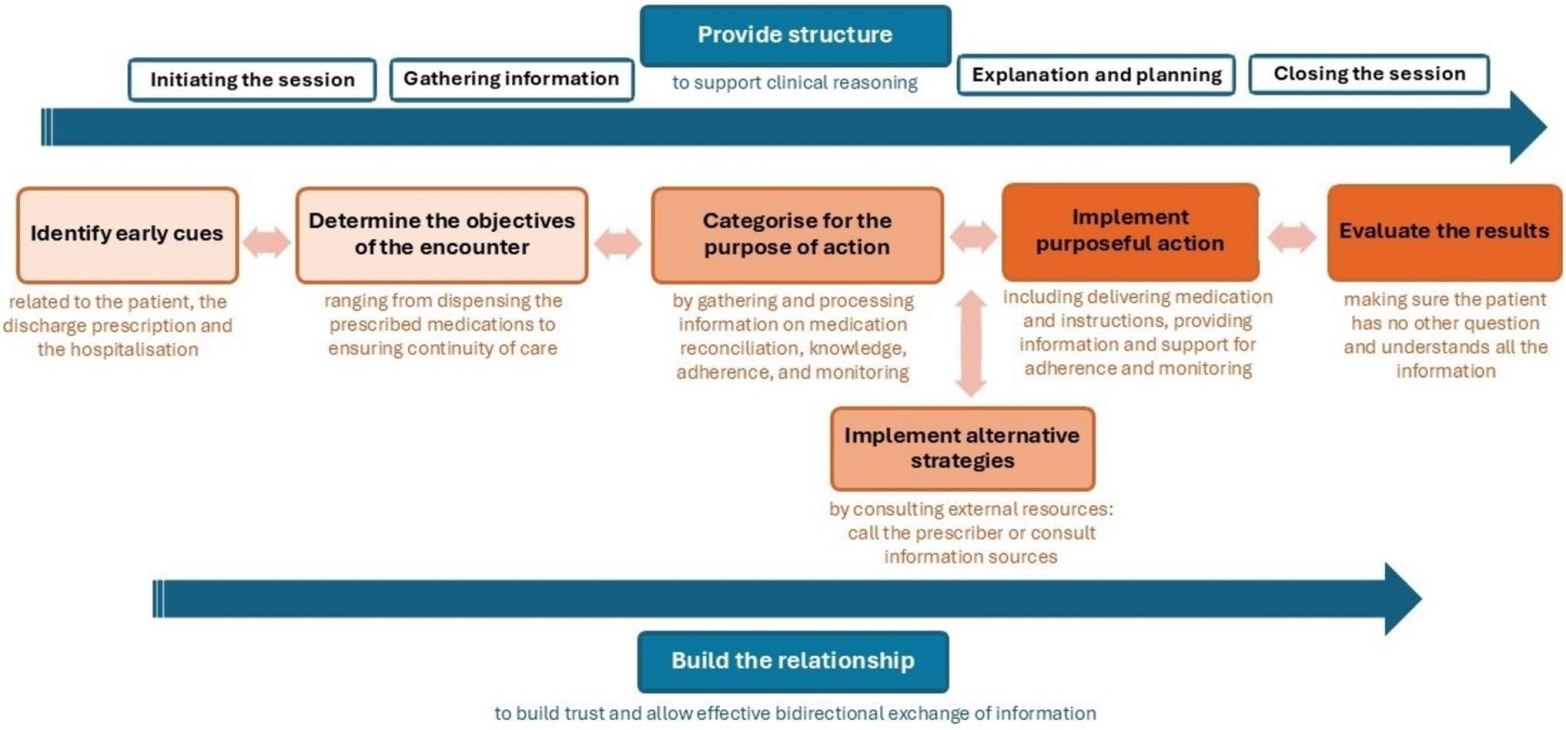
A combined model of the clinical reasoning model by Charlin et al. [13] (in orange) and the Calgary-Cambridge model [37] (in blue)

A combined model, integrating the six key steps of clinical reasoning according to Charlin et al. (in orange) and the two central concepts from the Calgary–Cambridge model (in blue), provides a detailed description of community pharmacists’ clinical reasoning when managing hospital discharge prescriptions, as illustrated in Fig. 2. Each step of the model is detailed below. Community pharmacists exhibited varying levels of clinical reasoning, which were influenced primarily by patient cues and the prescription context. The depth of clinical reasoning varied among community pharmacists, with all relying, at some point, on intuitive reasoning and assumptions, which introduced biases and impaired their clinical reasoning. Participants had a metacognitive analysis of their practices and their awareness of difficulties and potential biases in their clinical reasoning, as illustrated in Table 4.

**Table 4:** Clinical reasoning difficulties and potential cognitive bias

Identify early cues Difficulties in identifying cues	I didn’t really look at the patient and concentrated more on the prescription. […] What was her attitude, how was her walking, and was she overweighted? [rta12]
Determine the objectives of the encounter	
Difficulties in generating a reasoned objective	I didn’t think at all about the other peripheral aspects. I just gave the medications as prescribed. [rta 14]
Categorise for the purpose of action	
Difficulties in collecting data	I felt that this patient was completely overwhelmed by taking these medications. But I only found out at the end of the encounter. [rta13]
Poor representation of the clinical problem and anchoring bias^a^	I went straight to the prescription. Perhaps it would have been interesting to take a slightly higher point of view and not go straight to the [empagliflozin] that was introduced but see the whole treatment. [rta1]
Assumptions and premature closure	Looking at the medication history, I assumed that she was hospitalised for uncontrolled diabetes and increased hypertension. [rta6]
Omission bias^b^	As long as a patient doesn’t tell me or ask me about a side effect, […] I don’t get involved, and I don’t talk about it. [rta9]
Find alternative strategies	
Communication and time constraints	To keep it simple, I would have called the GP first […] For me, the hospital is a second choice because [I] don’t usually have direct numbers, so I waste a lot of time. [rta10]
Implement purposeful action	
Little consideration of the patient’s needs	When the prescription is long, I always recommend the pillbox straight away. The effect of a pillbox on medication adherence is amazing. [rta9]
Lack of shared understanding	I wasn’t sure what information she was waiting for. I could see she was waiting for some information, but it was unclear to me. [rta7]
Lack of shared decision-making	I decided to call the doctor to avoid making [the patient] wait too long, but I could have asked or proposed both alternatives, calling the doctor now or later. […] I should have taken better account of the patient’s needs. [rta5]
Evaluate the results	
Difficulty in getting an overall picture of the situation	At the end, I only asked if there were any other questions, I could have gone a little further by summarising, for example. [rta 13]
Provide structure	
Disorganised information gathering	I could have changed pravastatin to atorvastatin […] But I had already called the doctor [for medication reconciliation], and I would have had to call the doctor [again]. [rta6]
Lack of standardisation	In 15 min, you have to discuss different points, but you also have to make choices. As pharmacists, we don’t really have a standardised way to validate [a prescription]. [rt5]
Build the relationship:	
Difficulty in creating a climate of trust	Sometimes, patients are much more resistant to giving out information than him [= PP]. So, it’s more challenging to go into detail and obtain all the necessary information when this happens. [rta 11]
Patient centred care	She told me that she was not going to stay [in our pharmacy] and that she already has a usual pharmacy, so I went straight to the point and didn’t give too much information. [rta2]
Lack of time	It is not always possible to put the patient in the counselling room and take time to dive into all the problems. [rta12]

^a^Prematurely settling on a single problem based on the initial presentation; in this context, the pharmacist goes straight on medication rather than considering the broader context of the patient’s entire treatment regimen

^b^Choosing omission (inaction) over commission (action); in this context, the pharmacist avoids discussing potential side effects unless prompted by the patient

#### Identifying early cues

Some pharmacists adopted a passive approach, focusing primarily on basic demographic cues such as age:

“I just thought: an elderly person. I’m waiting to see what he has to say.” [retrospective think-aloud (rta) pharmacist 3].

In contrast, others employed a more active strategy, gathering multiple cues, including clinical context, medication profile, and physical appearance of the patient:

“It was a patient who had been discharged from the hospital with chronic medications, and he looked tired […] It was also a patient I didn’t know.” [rta8]

These clues helped the pharmacists build an initial representation of the problems, which in turn enabled some of them to anticipate drug-related problems and patient understanding issues.

#### Determine the objective of the encounter

Over half of the pharmacists articulated encounter objectives, ranging from accurate medication dispensing to ensuring continuity of care. The objectives of the encounter generally determined the topics covered, and community pharmacists examined various assumptions that guided their therapeutic decisions and actions. An example was to ensure patients understood and managed their medication regimen:

“The idea is for the patient to leave the pharmacy with all the information needed so that it is a treatment as simple as possible that can be followed daily.” [rta4]

#### Categorise for the purpose of action

Pharmacists categorised the actions by collecting patient information and cross-referencing it with prescriptions and medication history to identify Drug Related Problems (DRPs), including adherence and understanding issues. Most conducted medication reconciliation, addressing discrepancies by verifying comorbidities and medical history with the patient:

“On the prescription, [aspirin] was missing. I asked the patient if there had been a cardiovascular event that could justify the presence of aspirin. [...] I looked for cues […]to make sure that this medication was still necessary.” [rta13]

Several pharmacists actively assessed and took into account patients’ understanding of their medications, their ability to manage treatment, and patterns of non-adherence to previous regimens in order to tailor interventions and determine appropriate follow-up actions. While most pharmacists attempted to identify additional patient needs or questions at the end of the encounter, this approach often lacked structure and depth, resulting in important issues only emerging incidentally at the end of the consultation, leading the pharmacist back into a new categorisation:

“I asked if she had any other questions. And that’s when she told me that the evening intake was the problem. So, we had to find a solution.” [rta1]

#### Find alternative strategies

When faced with an unresolved problem, all pharmacists found alternative strategies. If the reason for changing a medication was unclear or not documented, pharmacists balanced legal requirements with their responsibilities, experience, and constraints. While pharmacists were expected to reach out to the prescriber to clarify intent proactively, the prescriber was often unreachable due to time constraints. Therefore, the pharmacist sometimes decided, under their responsibility and with careful documentation, to dispense the omitted medication. Another strategy involved utilising information technology resources to verify drug interactions, side effects, and prescribing instructions.

#### Implement purposeful action

Pharmacists employed a range of interventions based on the detected DRPs, including dispensing, providing medication instructions, discussing adherence, and assessing self-management. Many considered the availability of medication and follow-up timelines when dispensing the medications. Some pharmacists adapted their explanations to patient needs, simplifying complex information:

“I often try to simplify information. […] In this case, I explained the action of metformin by saying that it prevents the body from making sugar and that it limits the amount of sugar in the blood.” [rta6]

Side effects were seldom discussed unless the patient raised the topic. Approaches to supporting medication adherence varied: some pharmacists limited their interventions to providing pillboxes, while others adopted a more collaborative approach, engaging patients in shared decision-making to identify solutions that matched their preferences and lifestyle.

#### Evaluate the results

A small proportion of the pharmacists actively ensured that the patients understood the information by asking clarifying questions and requesting patient feedback, confirming their understanding of the key elements of their medication regimens.

#### Provide structure

Few pharmacists explicitly described a structured approach, such as reviewing each medication individually and addressing dosage and patient questions*.*Others reported adapting their approach and items based on the patient’s needs. Some emphasized the need for a more systematic method to enhance information exchange and understanding:

“The more information there is, the greater the risk of losing the person, so it’s important to practice synthesising.” rta1]

Unstructured information gathering resulted in information omissions, as described in Table 4. Experience may influence the structure, as senior pharmacists highlighted the lack of formal training in prescription validation (*“Nobody ever taught me how to validate a prescription. I do it on the go.”* [rta9]), while recent graduates reported using structured OSCE (Objective Structured Clinical Examination) frameworks (*“I just graduated, so the OSCE and the structured approach taught at the university are still strongly ingrained in my mind.”* [rta6]).

#### Build the relationship

Pharmacists highlighted the importance of establishing trust to gather essential information and foster a therapeutic alliance:

“My approach was to build a partnership with the patient, encouraging her participation and an open discussion. I also needed to involve her actively to maintain her interest.” [rta12]

The strategies for building trust and choosing communication styles often depended on the level of familiarity with the patient. Familiar patients allowed for more direct and personal engagement:

“If you know the patient before, it’s easier to say, ‘How are you doing? What happened?” […] If it’s someone you’ve never seen before, you have a bit more distance and respect for their private life, so the questions are a bit different.” [rta5].

### Clinical reasoning misconceptions

All community pharmacists encountered a variety of clinical reasoning misconceptions and biases, the majority of which were revealed through their metacognitive self-reflection on their professional actions. Table 4 outlines these difficulties and potential cognitive biases across each subprocess of Charlin’s model, with intuitive assumptions emerging as a critical factor in several key areas. Pharmacists often struggled to identify early patient cues and tended to focus more on the prescription than on the broader clinical context. Anchoring bias, premature closure, and omission bias were frequently observed.

## Discussion

### Statement of key findings

The simulated encounter with a discharged patient with type 2 diabetes and polypharmacy revealed varied community pharmacist practices. Pharmacists performed well in medication-related tasks but less in patient-centred activities, with inconsistencies in encounter structure and organisation.

Retrospective think-aloud interviews showed that pharmacists’ clinical reasoning varied, shaped by patient cues, prescription context, and encounter objectives. They navigated different hypotheses and all encountered reasoning misconceptions, such as assumptions and premature closure, affecting decisions. Participants also demonstrated metacognitive awareness of their practices and potential biases.

### Strengths and weaknesses

The simulated encounter in the participants’ own pharmacy, combined with immediate retrospective think-aloud interviews, provided insights into real-world community pharmacy practices while also capturing pharmacists’ decision-making processes when faced with a complex prescription. One strength of this study lies in its rigorous qualitative methodology. Data saturation was actively pursued and achieved. To enhance trustworthiness, multiple investigators with expertise in clinical reasoning coded the data independently and met to reach consensus. Furthermore, our analysis, guided by the Charlin et al. transdisciplinary clinical reasoning model [13], is the first to apply this framework to community pharmacists’ clinical reasoning. The study provides key insights into both community pharmacists’ and clinical reasoning, offering valuable contributions to the limited body of literature on this subject.

Limitations include potential selection bias, as participating pharmacists were likely more engaged than average. Additionally, advanced scheduling of visits may have led to practices that reflect optimal rather than routine behaviour. Second, pharmacists’ descriptions of their clinical reasoning were at times superficial and primarily focused on observable actions, rather than on the explicit articulation of the underlying cognitive processes. This tendency may be partly explained by the non-existent training pharmacists receive in clinical reasoning, both at the undergraduate and postgraduate levels. Although data saturation was achieved in the qualitative analysis, saturation was not reached in the quantitative data.

### Interpretation

During simulations, pharmacists processed hospital discharge prescriptions in a median of 20 min (IQR: 18–21; range: 9–38), longer than the average prescription encounter (8.5 min) but consistent with reported durations for discharge prescriptions (10–40 min) [42]. This reflects the greater complexity and need for clinical interventions to ensure patient safety during care transitions [41, 42]. Pharmacists from our study prioritised medication-related tasks over patient-centred activities, and this aligns with existing literature [41, 43]. This focus on technical tasks likely reflects professional habits, time pressures, workflow demands, and greater confidence compared to more complex patient-centred activities [44]. During care transitions, reinforcing patient-centred activities, such as patient education, follow-ups, and home visits in crucial to improve care transitions and reduce hospital readmissions [45, 46]. Additionally, the frequent contacts of pharmacists with patients at discharge and during regular follow-ups allows them to address drug-related problems, such as unintentional nonadherence and mitigate barriers, such as low health literacy and limited access to care [24, 47–49]. However, these skills remain under-recognised and under-reimbursed, limiting integrated patient-centred care in outpatient settings [21, 36]. Promoting pharmaceutical care, standardising practices and fostering interprofessional collaboration at the hospital‒community interface are therefore essential [50, 51].

Through retrospective think-aloud interviews, our results indicated that community pharmacists exhibited varying levels of clinical reasoning. Our results converge with other studies, showing that pharmacists use multiple cognitive processes, frequently revisiting hypotheses and therapeutic options to assess medication appropriateness and safety [16, 52, 53]. Pharmacists in our study used more analytical and structured reasoning for medication-related than patient-related problems, consistent with scoping review findings [5]. Our results revealed biases and clinical reasoning misconceptions throughout pharmacists’ clinical reasoning, such as assumptions leading to premature closure, challenges also seen in other healthcare professionals [39, 54, 55]. Established strategies are available to reduce clinical reasoning deficits and support learners in refining their decision-making processes [56, 57], which should be included in pharmacy pre- and postgraduate training [58].

In this study, the Charlin et al. model was applied for the first time to analyse community pharmacists’ clinical reasoning and was previously been used for general practitioners and hospital pharmacists [59, 60]. Although hospital-based clinical pharmacists and community pharmacists employ similar reasoning processes in their clinical activities [60], limited access to patient information and interaction with other practitioners seem to be barriers to effective reasoning. These findings highlight the need to improve information flow, for example, by integrating pharmacists into care teams or enabling access to electronic health records or a pharmaceutical hotline.

We integrated Charlin et al.’s clinical reasoning model [13] with the Calgary-Cambridge encounter structure model [37] to illustrate the interplay between communication, structure of the interview, and clinical reasoning. Community pharmacists in our study often revisited various themes, deviating from the Calgary–Cambridge Model, leading to inconsistencies and missed information, that may impact their clinical reasoning. Adopting a semi-structured, evidence-based consultation model would provide a reliable foundation for less experienced pharmacists, helping ensure comprehensive care and reducing omissions, while allowing for flexibility to meet individual needs. Experienced pharmacists may adapt the structure more flexibly, drawing on their mastered cognitive frameworks to organise and complete information gathering [56, 61].

### Future research and implication for practice

Future research should apply the Charlin et al. clinical reasoning model [13] to various pharmaceutical care contexts and prescription scenarios. Enriching future research with complementary methods, such as reviewing pharmacists’ encounters and using open-ended, reasoning-focused questions, could encourage more in-depth reflection and help capture the full complexity of their clinical decision-making.

Guidelines and training should equip community pharmacists with skills to manage medication and patient challenges after hospital discharge by combining clinical reasoning, medication reconciliation, and patient involvement. An integrative, competency-based yet flexible approach could help structure encounters and ensure patient needs are addressed.

Integrating clinical reasoning into pharmacy education will help pharmacists critically evaluate practices, recognise biases, and make informed decisions. Clarifying their roles in interprofessional teams will further enhance patient care and healthcare system efficiency.

## Conclusion

This study highlighted that community pharmacists perform well in medication-related tasks but engage less in patient-centred activities, with inconsistencies in interview structures and clinical reasoning approaches. Community pharmacists relied on both analytical and intuitive reasoning, often influenced by cognitive biases, which has an impact on decision-making and patient care. Our findings highlight the urgent need for undergraduate and postgraduate training in clinical reasoning.

## Supplementary Information

Below is the link to the electronic supplementary material.

Supplementary file1 (DOCX 16 KB)

Supplementary file2 (DOCX 17 KB)

Supplementary file3 (DOCX 25 KB)

Supplementary file4 (DOCX 137 KB)
