# Supplementary material for: Community pharmacists’ practices and clinical reasoning towards hospital discharge prescription: a study using simulations and retrospective think-aloud methodology
Source: Int J Clin Pharm. 2025 Aug 26;48(1):127–38. doi: 10.1007/s11096-025-01978-0 (PMC12823730; doi:10.1007/s11096-025-01978-0)
Supplement: Supplementary file 1 — Supplementary file1 (DOCX 16 KB) [file 11096_2025_1978_MOESM1_ESM.docx]

**ELECTRONIC SUPPLEMENTARY MATERIAL 2 : The pharmacists’ practice checklist**

| **Themes** | Items |
| --- | --- |
| **1. Legal validity & administration tasks** | Check the prescription validity and recipient; Create patient records; Validate insurance cards; and others. |
| **2. Medical history** | Check the reason for the prescription; Check for comorbidities; Check allergies; Check and complete the patient's medication history (including OTC and alternative medicine); Identify the new medication; Identify medication changes (changes in dosage, omission, generic switch and dosage); Identify changes in medication timing (morning, evening); others. |
| **3. Prescription validation** | Check for the absence of drug interactions and contraindications; Intervene regarding medication omission (e.g. Contact the prescriber to verify medication discrepancies); Switch between original and generic medication; Check for the next medical and pharmacy appointments and define the size of the medication boxes accordingly; others |
| **4. Medication dispensing** | Check medications needed; Suggest alternatives when medication is unavailable (e.g. order medication, supply from another pharmacy); Provide the medications needed and label with instructions on each package; Explain the medication regimen and instructions; others. |
| **5. Patient medication knowledge** | Check patient knowledge about medications; Identify a lack of knowledge about the new medication; Provide information on medications; Identify the need for information about potential side effects; Provide information on side effects how to prevent and manage them; others. |
| **6. Medication management and adherence** | Evaluate previous experience with medication management at home; Suggest ways to manage medication; Assess medication adherence; Identify medication adherence difficulties, particularly in the evening; Consider the patient's opinion, needs, and available resources; Informe on medication management (e.g. routine development, medication plan, weekly planner, therapeutic alternatives); others |
| **7. Monitoring** | Check the understanding of information discussed during the encounter (teach-back method); Check self-monitoring skills (e.g. blood & glucose monitoring); Check warning signs of clinical deterioration are known (e.g. hypoglycaemia values); Liaise with the general practitioner; others. |
