# Supplementary material for: Community pharmacists’ practices and clinical reasoning towards hospital discharge prescription: a study using simulations and retrospective think-aloud methodology
Source: Int J Clin Pharm. 2025 Aug 26;48(1):127–38. doi: 10.1007/s11096-025-01978-0 (PMC12823730; doi:10.1007/s11096-025-01978-0)
Supplement: Supplementary file 2 — Supplementary file2 (DOCX 17 KB) [file 11096_2025_1978_MOESM2_ESM.docx]

**ELECTRONIC SUPPLEMENTARY MATERIAL 1: INTERVIEW GUIDE - Retrospective think-aloud**

Thank you for participating in this simulation. In the second phase, we aim to capture and analyse your thoughts and decision-making process during the prescription validation. Together, we will review the steps you followed in the simulation to better understand your reasoning and approach

As a reminder, all this data will be anonymised. #:

| **General opening question** | From the moment the patient arrives at the counter, describe what were your thoughts/ what you looked at ? |
| --- | --- |
| *Follow-up question* | Okay, continue to explain your reasoning.  What did you think of next? |
| *A question of precision* | At this moment, xxx, why did you say xxx?  What made you think of this?  How did you react to this answer? |
| *If the person is blocked* | To help you, you can think about what you would explain to a new pharmacist trainee. |
| **Simulation milestones** | *Opening and information gathering* |
|  | *Identify the problem with medication reconciliation and take appropriate action* |
|  | *Medication dispensing* |
|  | *Patient understanding of medication and action taken* |
|  | *Medication adherence and action* |
|  | *Monitoring* |
|  | *Closing the interview* |
| **Closing question** | How did this simulation differ from your usual practice? |
|  | What did you think of the simulation? |
|  | How might this reflection influence your practice? |

Duration :

Location: consultation room, back office, office, other :

Field note :
