# Supplementary material for: Community pharmacists’ practices and clinical reasoning towards hospital discharge prescription: a study using simulations and retrospective think-aloud methodology
Source: Int J Clin Pharm. 2025 Aug 26;48(1):127–38. doi: 10.1007/s11096-025-01978-0 (PMC12823730; doi:10.1007/s11096-025-01978-0)
Supplement: Supplementary file 3 — Supplementary file3 (DOCX 25 KB) [file 11096_2025_1978_MOESM3_ESM.docx]

| **ELECTRONIC SUPPLEMENTARY MATERIAL 3: Items raised at least once by participants during the simulation** | | |
| --- | --- | --- |
| **Main themes** | **Steps** | **n/14** |
| Prescription validity and administration | Check the prescription validity and recipient | 11 + 3NA |
|  | Creat patient records and validating insurance card | 14 |
| Medical history | Check allergies | 6 |
|  | Check for co-morbidities | 6 |
|  | Check the reason for the prescription | 10 |
|  | Check and complete medication history (including OTC and alternative medicine); | 13 |
|  | Identify the new medication (empagliflozin) | 13 |
|  | Identify changes in dosage (valsartan, metformin) | 13 |
|  | Identify the lack of medication on the prescription (aspirin) | 12 |
|  | Identification of a generic switch | 7 |
| Prescription validation | Medication reconciliation (Aspirin): intervention undertaken :   - dispensing aspirin following contact with the doctor - Non-dispensing of aspirin cardio - Dispensing aspirin cardio without prescriber’s approval | 10  2  2 |
|  | Generic (Escitalopram): Switch to original | 7 |
|  | Check for drug interactions and contraindications | 7 |
|  | Check for the next medical and pharmacy appointments and define the size of the medication boxes accordingly | 9 |
| Medication dispensing | Check the medications needed | 4 and 3NA |
|  | Suggest alternatives when medication is unavailable | 8 |
|  | Provide the medications needed and label with instructions on each package | 14 |
|  | Explain the medication regimen and instructions | 14 |
| Medication knowledge | Check patient knowledge about medications | 2 |
|  | Identify a lack of knowledge about the new medication (empagliflozin) | 4 of which 1*. |
|  | Provide information on medications | 12 of which 1* |
|  | Identify the need for information about potential side effects | 2 |
|  | Provide information on side effects how to prevent and manage them | 8 of which 6*. |
| Medication management | Evaluate previous experience with medication management at home | 6 |
|  | Suggest ways to manage medication (e.g. bringing back old boxes) | 4 |
| Medication adherence | Assess the degree of (non)adherence | 7 |
|  | Identify medication adherence difficulties, particularly in the evening | 9 of which 3* |
|  | Consider the patient's opinion, needs, and available resources | 5 |
|  | Informe on medication management   - Medication pillbox - Treatment plan - Therapeutic alternative - Routine development | 11  7  3  2  2 |
| Monitoring | Check self-monitoring skills (e.g. blood & glucose monitoring) | 9 |
|  | Check warning signs of clinical deterioration are known (e.g. hypoglycaemia values) | 2 |
|  | Check the understanding of information discussed during the encounter (teach-back method). | 2 |
| Others | Continuity of care with the patient's pharmacy  Liaise with the general practitioner  Tolerance and need of long-term medication  Loyalty card | 4  4  3  2 |

NA: non-applicable

*discussed by the patient after the participant says: "Do you have any questions?"
