# Supplementary material for: Community pharmacists’ practices and clinical reasoning towards hospital discharge prescription: a study using simulations and retrospective think-aloud methodology
Source: Int J Clin Pharm. 2025 Aug 26;48(1):127–38. doi: 10.1007/s11096-025-01978-0 (PMC12823730; doi:10.1007/s11096-025-01978-0)
Supplement: Supplementary file 4 — Supplementary file4 (DOCX 137 KB) [file 11096_2025_1978_MOESM4_ESM.docx]

| **Y-axis**: 1. Prescription validity and admin, 2. Medical history 3. Prescription validation 4. Medication dispensing, 5. Medication Knowledge, 6. Medication management and adherence / 7. Monitoring  **X-axis**: order of occurrence of the items  ***Thematic order:** according to our Community Pharmacists Practice Checklist | **Y-axis:** 1. Initiating the session; 2. Gathering information 3. Explanation and planning; 4. Closing the session  **X-axis:** order of occurrence of steps |
| --- | --- |
|  |  |
|  |  |
|  |  |
|  |  |
|  |  |
|  |  |
|  |  |
|  |  |
|  |  |
|  |  |
|  |  |
|  |  |
|  |  |
|  |  |
